# Resolving the Texture–Flavor Trade-Off in ‘Annurca’ Apples with an Integrated Postharvest System

**DOI:** 10.3390/foods14203554

**Published:** 2025-10-18

**Authors:** Giandomenico Corrado, Alessandro Mataffo, Pasquale Scognamiglio, Maurizio Teobaldelli, Boris Basile

**Affiliations:** Department of Agricultural Sciences, University of Naples Federico II, 80055 Portici, Italy; giandomenico.corrado@unina.it (G.C.); alessando.mataffo@unina.it (A.M.); pasquale.scognamiglio2@unina.it (P.S.); maurizio@teobaldelli.eu (M.T.)

**Keywords:** *Malus domestica*, sensory analysis, consumer preference, postharvest technology, consumer segmentation

## Abstract

The ‘Annurca’ apple, a traditional Italian cultivar protected by the “Melannurca Campana” EU PGI designation, undergoes a mandatory, traditional postharvest reddening process in a melaio. While essential for developing its characteristic flavor and color, this process can also lead to significant textural degradation, resulting in a mealy and soft fruit that conflicts with modern consumer expectations. This study investigated an integrated postharvest strategy to resolve this quality trade-off. We evaluated the sensory profile and consumer acceptance of ‘Annurca’ apples subjected to three treatments: traditional melaio reddening (Melaio), a 1-methylcyclopropene treatment alone (MCP), and a combined treatment of MCP followed by melaio reddening (MCP+Melaio). A panel of 534 consumers evaluated the apples for overall liking and the intensity of seven key sensory attributes. The results showed that the integrated ‘MCP+Melaio’ treatment was significantly preferred (Mean liking = 6.61) over both the traditional ‘Melaio’ (M = 5.91) and ‘MCP’ alone (M = 5.91) treatments. This preference was driven by a superior sensory profile that combined the high crunchiness and low mealiness of the MCP treatment with the high perceived aroma intensity and sweetness developed during the melaio phase. Furthermore, consumer segmentation analysis identified four distinct preference clusters, revealing that the integrated treatment’s success derived from its ability to satisfy the divergent priorities of the two largest segments: “Melaio Fans” (37%) and “Texture & Flavor Seekers” (35%). Our findings demonstrate that combining 1-MCP with traditional practices creates a synergistic effect, producing a high-quality apple that is texturally superior, aromatically intense, and has an extended sensory shelf-life. This integrated approach offers a scientifically validated and practical solution to enhance the quality and consistency of ‘Annurca’ apple production.

## 1. Introduction

The apple (*Malus domestica*) is one of the most economically significant and widely consumed fruits globally, with consumer preference and market value being largely dictated by its quality [1,2]. Consumer acceptance is primarily driven by a combination of textural attributes, such as crispness, hardness, and juiciness, and a satisfying flavor profile, defined by a balance of sweetness, acidity, and the presence of characteristic aroma compounds [3]. Given the seasonal nature of apple production, postharvest storage in controlled conditions is nowadays a standard, indispensable procedure for meeting year-round consumer demand and ensuring market availability worldwide.

Among the vast apple germplasm [4], certain varieties hold unique cultural and economic significance due to their distinct organoleptic profiles and traditional production methods [5]. A prime example is the ‘Annurca’ apple, an ancient cultivar from Southern Italy locally acclaimed as the “Queen of Apples”, and its clonal derivatives like ‘Rossa del Sud’ [6,7]. This fruit is protected by the “Melannurca Campana” EU Protected Geographical Indication (PGI) designation, underscoring its deep connection to the territory of origin. A defining element of its PGI specification is the mandatory postharvest reddening process (Reg. EU No 417/2006), carried out in the *melaio*. This is a bed of wood chips (or, more traditionally, hay) where the apples are laid out for several weeks to develop their characteristic ruby-red skin [8]. Despite its cultural importance, the traditional *melaio* system presents a fundamental technological contradiction. The extended reddening period at ambient temperatures allows for uncontrolled ethylene biosynthesis and action. While this ethylene-driven ripening process is essential for developing the fruit’s characteristic ruby-red skin and desirable aromatic profile [8,9], it simultaneously accelerates senescence. This leads to a significant and inconsistent degradation of fruit texture, primarily characterized by a loss of firmness and an undesirable mealy quality. This textural failure places the ‘Annurca’ apple at a competitive disadvantage against modern, uniformly crisp cultivars [10], creating a critical trade-off between flavor development and textural integrity.

To counteract senescence-related softening, the use of 1-methylcyclopropene (1-MCP) has become a widely adopted and effective strategy in the global apple industry [11]. This synthetic compound acts as a strong inhibitor of ethylene perception, delaying ripening and senescence by blocking the hormone’s signal transduction pathways, thereby preserving firmness and extending the shelf life of many apple cultivars [12,13]. However, its application to *Malus domestica* ‘Annurca’ poses a significant challenge placing the ‘Melannurca Campana’ industry at a critical intersection, with the need to balance a traditional postharvest method that adversely affects texture and a contemporary treatment that diminishes the fruit’s characteristic flavor.

To address these issues, our study investigated the impact of traditional postharvest reddening in the *melaio*, the application of 1-MCP, and a combined approach (1-MCP followed by *melaio* reddening) on the quality attributes of ‘Rossa del Sud’ apples, the clonal derivative of ‘Annurca’ that currently dominates the PGI Annurca production. This work builds upon previous research, which explored the integration of 1-MCP into the Annurca’s traditional reddening system, demonstrating its potential to positively affect fruit flesh firmness and sensory attributes [8].

Since consumer acceptance plays a decisive role in product success, a comprehensive sensory evaluation was conducted with a large consumer panel to assess overall liking and specific textural and flavor attributes. Our experimental design allowed for a direct comparison of the traditional method’s effects on texture and flavor, the isolated impact of 1-MCP on these parameters, and the potential of combined treatment to mitigate textural degradation while preserving the characteristic sensory profile. Specifically, we aimed to elucidate the interplay between postharvest treatments, sensory perception, and consumer preferences using linear mixed-effects models and consumer segmentation to provide critical insights into the future of ‘Melannurca Campana’ production.

## 2. Materials and Methods

### 2.1. Plant Material and Postharvest Treatments

Following the traditional harvest protocol, fruits of the apple (*Malus domestica*) cultivar ‘Rossa del Sud’, a clonal derivative of ‘Annurca’, were handpicked before full skin color development from a commercial orchard in the Campania region, Italy (Giaccio Frutta Soc. Coop., Vitulazio (CE), Italy). Apples were harvested under the production protocol for the “Melannurca Campana”. Immediately after harvest, around 4500 apples of the commercial size diameter class 65–85 mm were divided into three groups to undergo different postharvest treatments: (1) ‘Melaio’ (Control): Fruits were subjected to the traditional reddening process. Briefly, apples were arranged in a single layer on woodchip beds in an open field structure (“melaio”) protected by shading nets and manually rolled over for 10 days to ensure uniform skin reddening. Then fruit were stored in a commercial cold room set at a temperature of 1 °C and a relative humidity of 90%, equipped with an ethylene extractor; (2) ‘MCP’: Fruits were pre-cooled, placed in commercial air-tight cold room (set at a temperature of 2.5 °C), and treated with a commercial product (Smartfresh™, Agrofresh, Philadelfia, PA, USA) generating 1-methylcyclopropene (1-MCP) gas at 55.5 mg/m^3^ for 24 h. After treatment, apples were stored as described for the ‘Melaio’ treatment; (3) ‘MCP+Melaio’: Fruits were first treated with 1-MCP as described for the ‘MCP’ group and subsequently underwent the traditional melaio reddening process and cold storage as described for the ‘Melaio’ treatment. Sensory evaluation sessions were conducted on seven dates over a period spanning from 0 to 179 days of cold storage.

### 2.2. Participants

A total of 558 consumers participated in the study, all recruited from the Department of Agricultural Sciences at the University of Napoli Federico II, Italy. The sensory evaluation was conducted in a dedicated room, maintained at a controlled temperature (20–22 °C) and ventilated to prevent the accumulation of aromas. Testing took place in individual booths equipped with uniform, white lighting to ensure that sample appearance was judged consistently and without distraction. The panel consisted of 208 female and 326 male participants, while 24 chose not to disclose their gender. In terms of age, 471 participants were aged 30 years or younger, and 59 were over 30 years old; 28 participants preferred not to report their age. After data cleaning (Section 2.4.1), the final dataset included 534 consumers for the main statistical analyses. Criteria for inclusion required participants to be regular apple consumers, defined as consuming apples at least once a week, and to have no known allergies to apples. All participants were provided with information about the study and gave their informed consent prior to the evaluation.

### 2.3. Sensory Evaluation Procedure

Sensory tests were conducted over seven dates, with each session taking place in the morning. On each date, participants were organized into sub-sessions of approximately ten people. The experimental design ensured that each consumer evaluated a sample from all three treatments. The presentation order of the samples was balanced and randomized across participants to minimize order and carry-over effects. Fruit samples were prepared immediately before testing. The use of anti-browning agents was deemed unnecessary, as the short time between preparation and evaluation effectively prevented discoloration and ensured that panelists assessed the fruit’s unaltered sensory characteristics. Two longitudinal slices (approx. 30 mm wide) were cut from each apple, with the skin left on. Samples were presented on a tray in disposable plastic cups labeled with random three-digit codes. Consumers were asked to taste each sample and rate their Overall Liking on a 9-point hedonic scale (1 = extremely dislike; 5 = neither like nor dislike; and 9 = extremely like). They were also asked to rate the intensity of the following sensory attributes: Hardness, Crunchiness, Juiciness, Mealiness, Sweet Taste, Sour Taste, and Aroma Intensity, using a 9-point scale (1 = very low; 5 = medium; and 9 = very high).

### 2.4. Statistical Analysis

All statistical analyses were performed using R (Version 4.3) running in RStudio build 421 (Rstudio, Boston, MA, USA). Briefly, data manipulation, transformation, and visualization were conducted using packages from the tidyverse suite (e.g., dplyr, tidyr, ggplot2) [14]. A significance level of α = 0.05 was used for all statistical tests. To identify each participant across different testing dates, a unique identifier (Unique_Consumer_ID) was created by combining the consumer ID with the testing date.

#### 2.4.1. Analysis of Main Effects and Interaction

To assess the overall effect of the treatments, a linear mixed-effects model (LMM) was fitted using the lmerTest package [15]. Overall Liking was modeled as the dependent variable with Treatment as a fixed effect. To account for participant-level variability in scoring, the Unique_Consumer_ID was included as a random effect. To account for potential session-to-session variability, the testing Date was also included as a random effect. A separate LMM was used to test for the interaction Treatment × Storage Time, with storage duration (in days) treated as a continuous numeric predictor. For all models, the significance of fixed effects was determined using Type III ANOVA with Satterthwaite’s method for degrees of freedom as a computationally efficient way to approximate the degrees of freedom and calculate *p*-values for the fixed effects in a linear mixed-effects model. The LMM approach handled missing data by using all available information for each specific analysis; a participant’s data was excluded from a given model if a value was missing for one of the specific variables included in that particular model. Post hoc pairwise comparisons for significant effects were conducted using the emmeans package, with *p*-values adjusted via the Tukey method.

#### 2.4.2. Consumer Segmentation by Preference

To identify segments of consumers with distinct preference patterns, a hierarchical cluster analysis (HCA) was performed. The HCA was performed on a consumer-by-treatment matrix using the squared Euclidean distance as the dissimilarity measure and Ward’s D2 agglomeration method, which minimizes within-cluster variance [16]. The optimal number of clusters was determined by visually inspecting the dendrogram and assessing the average silhouette width and the within-cluster sum of squares (“elbow” method), using the factoextra package [17].

#### 2.4.3. Characterization and Profiling of Consumer Segments

Following segmentation, each cluster was characterized demographically and by its unique sensory drivers. The association between cluster membership and demographic factors (Age, Gender) was evaluated using Pearson’s Chi-squared (χ^2^) test of independence. To confirm that the segments had statistically different preferences, a one-way ANOVA followed by Tukey’s HSD test was performed on the Overall Liking scores within each cluster using the agricolae package [18]. Sensory Profiling and Driver Analysis were carried out following a two-step process to understand the sensory basis for each segment’s preferences. First, the sensory profile for each cluster was described by calculating the mean ratings of the seven sensory attributes for each treatment. Second, to quantify the specific attributes driving preferences, a separate LMM was fitted for each cluster’s data subset. In these models, Overall Liking was the dependent variable, the seven sensory attributes were fixed effects, and Unique_Consumer_ID and Date were included as random effects, mirroring the structure of the main effects model.

## 3. Results

### 3.1. Main Effect of Postharvest Treatment on Overall Liking

A linear mixed-effects model revealed a significant main effect of the postharvest treatment on consumer overall liking (F(2, 1061.3) = 28.99, *p* < 0.001). To dissect this overall effect, post hoc pairwise comparisons using the Tukey method were performed on the estimated marginal means.

**Figure 1:**
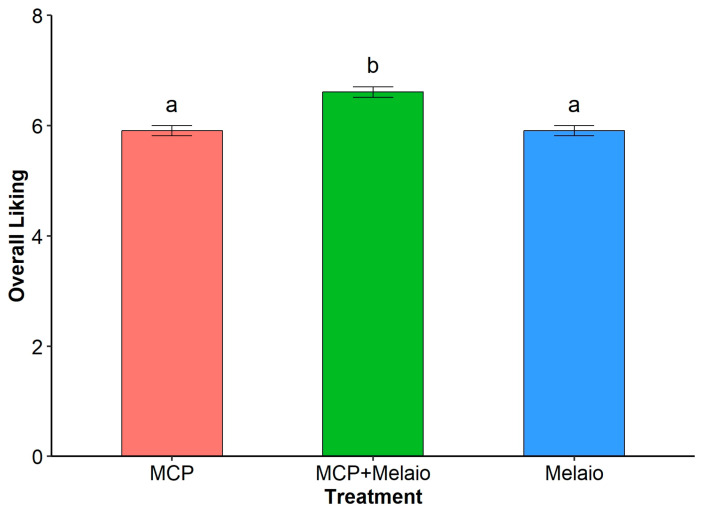
**Effect of postharvest treatments on consumer overall liking scores.** Bars represent the estimated marginal means from a linear mixed-effects model. Error bars represent the standard error of the mean. Bars with different letters are significantly different from each other based on Tukey’s HSD post hoc test (*p* < 0.05).

The analysis showed that the combined MCP+Melaio treatment resulted in the highest liking scores (Mean = 6.61, Standard Error = 0.095). These scores were significantly higher than those for the MCP treatment alone (M = 5.91, SE = 0.091; *p* < 0.001) and the Melaio treatment alone (M = 5.91, SE = 0.091; *p* < 0.001). The liking scores for the MCP and Melaio treatments were statistically indistinguishable from one another (*p* = 0.999) (Figure 1).

The analysis of mean sensory scores provides a solid explanation for the observed overall preference among treatments (Table 1). The combined ‘MCP+Melaio’ treatment emerged as the most effective, offering the most balanced sensory profile. This integrated approach resulted in the highest Aroma Intensity rating (5.54), significantly surpassing that of apples treated with 1-MCP alone, but not being different from the ‘Melaio’ treatment. In addition, the combined treatment preserved excellent textural attributes, with significantly lower mealiness (3.38) and higher crunchiness (6.29) compared to fruit subjected to the traditional ‘Melaio’ method. Although apples from the ‘Melaio’ treatment were perceived as the sweetest, their inferior texture (evidenced by the highest mealiness and lowest crunchiness scores) diminished this advantage. The combined enhancement in both aroma and texture likely accounts for the superior overall liking of MCP+Melaio apples.

**Figure 2:**
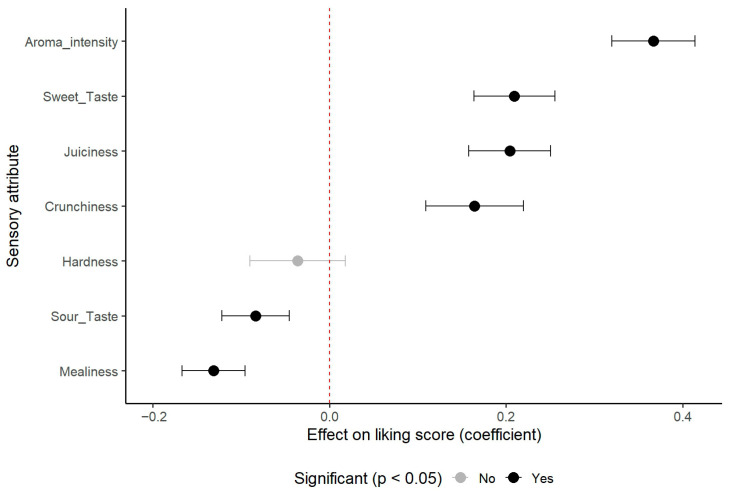
**The effect of sensory attributes on consumer overall liking as determined by a linear mixed-effects model.** The plot displays the coefficient estimates (β; dots) and their corresponding 95% confidence intervals (horizontal bars) for each attribute. The vertical dashed line at zero indicates no effect. Attributes whose confidence intervals do not cross the zero line have a statistically significant positive (to the right of the line) or negative (to the left of the line) impact on the final overall liking score.

To understand the sensory drivers behind these preferences, the relationship between individual sensory attributes and Overall Liking was modeled for the entire consumer panel. The analysis revealed that Aroma Intensity was the most powerful positive predictor of consumer liking (β = 0.37, *p* < 0.001), as visually summarized in Figure 2.

Other significant positive drivers included Sweet Taste (β = 0.21, *p* < 0.001), Juiciness (β = 0.20, *p* < 0.001), and Crunchiness (β = 0.16, *p* < 0.001). In contrast, Mealiness was a strong negative predictor (β = −0.13, *p* < 0.001), as was Sour Taste (β = −0.08, *p* < 0.001). Hardness was not found to have a significant effect on Overall Liking (*p* = 0.191) after accounting for the other textural attributes.

**Figure 3:**
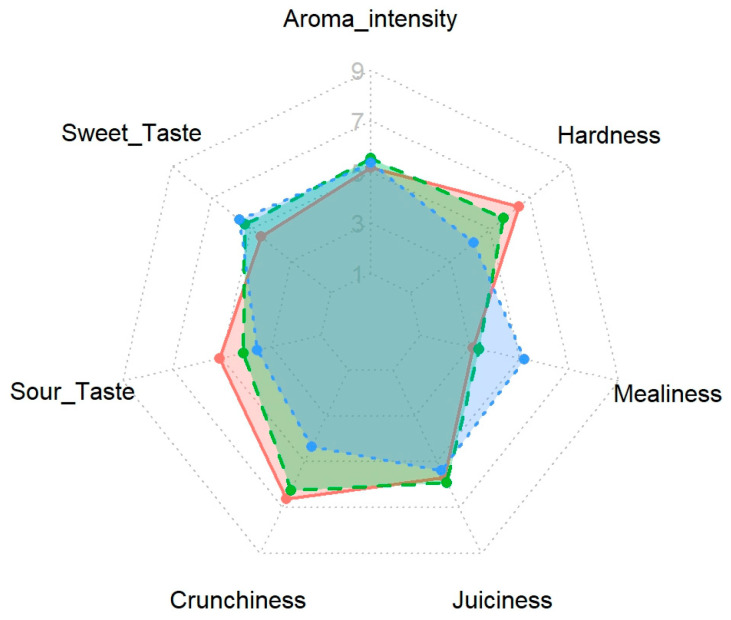
**Sensory profiles of ‘Annurca’ apples subjected to three different postharvest treatments.** Values are mean consumer ratings on a 9-point intensity scale. Treatments shown are ‘Melaio’ (blue dashed line), ‘MCP’ (salmon solid line), and ‘MCP+Melaio’ (green dot-dashed line).

Given the relevance of these sensory attributes, we next analyzed the effect of the postharvest treatments on the apples’ sensory profiles. As visualized in the spider plot in Figure 3, the treatments produced distinctly different sensory fingerprints, highlighting a clear trade-off between textural and flavor attributes. Specifically, the profile of the traditional ‘Melaio’ treatment (blue line) was characterized by a high score in Sweet Taste and Mealiness but a notably low score in Crunchiness. Conversely, the ‘MCP’ treatment (salmon line) showed the opposite profile, dominated by high scores for textural attributes like Crunchiness and Hardness at the expense of lower Sweet Taste and the lowest Aroma Intensity. The integrated ‘MCP+Melaio’ treatment (green line) demonstrated the most desirable and balanced profile. Crucially, this treatment produced the highest rated Aroma Intensity, while also successfully combining high Crunchiness with a well-developed Sweet Taste and low Mealiness.

In summary, the consumer preference for the ‘MCP+Melaio’ treatment is explained by its uniquely superior sensory profile. Crucially, this integrated treatment produced the highest-rated Aroma Intensity, the single most important positive driver of liking, while also developing a significantly higher Sweet Taste compared to the ‘MCP’ apples. Simultaneously, it successfully mitigated the negative textural qualities of the traditional process, delivering significantly lower Mealiness and higher Crunchiness than the ‘Melaio’ treatment. This demonstrates that the combined treatment created an apple that was aromatically intense, flavorful, and texturally crisp, aligning very well with all the key drivers of consumer preference identified in our model and explaining its top ranking in Overall Liking.

### 3.2. Effect of Storage Time and Its Interaction with Treatment

**Figure 4:**
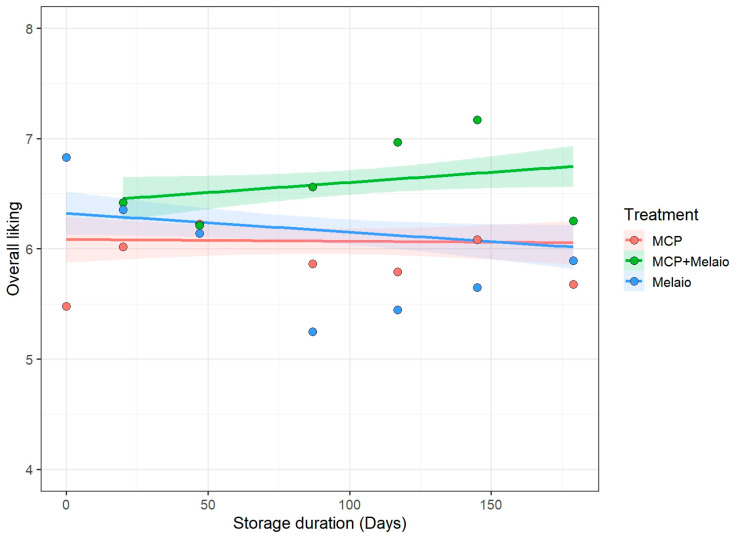
Interaction between postharvest treatment and storage time on consumer overall liking. Liking for the traditional ‘Melaio’ treatment (blue) declined over time, while ‘MCP’ (salmon) and ‘MCP+Melaio’ (green) remained more stable. Solid lines show model predictions (95% CI shaded); points indicate mean scores per date.

To assess the impact of storage duration on consumer preference, the interaction between postharvest treatment and storage time was analyzed. The analysis revealed a highly significant Treatment × Storage Day interaction (F(2, 1106.2) = 7.89, *p* < 0.001), indicating that the relative performance of the treatments changed significantly over the storage period. As visualized in Figure 4, the liking scores for apples from the traditional ‘Melaio’ treatment declined at a considerably faster rate than those for the two treatments involving 1-MCP application. ‘MCP’ apples showed a very stable liking score over time, while the ‘MCP+Melaio’ fruit showed an increase in consumer acceptance. In contrast, the traditional method induced a product of diminishing quality, providing strong evidence that a key benefit of incorporating 1-MCP is a significant extension of the apple’s sensory shelf-life.

### 3.3. Influence of Consumer Demographics

To investigate whether the observed treatment effects were consistent across different consumer segments, the main linear mixed-effects model was extended to test for interactions with age and gender. The analysis revealed no significant interaction between postharvest treatment and consumer age group on Overall Liking scores (F(2, 1028.0) = 1.86, *p* = 0.156). This indicates that the relative preference for the treatments was consistent across both younger (≤30 years) and older (>30 years) consumer groups. Similarly, no significant interaction was found between treatment and consumer gender on Overall Liking scores (F(2, 1033.4) = 0.95, *p* = 0.386). This implies that both male and female participants responded comparably to the treatments. Therefore, the main findings regarding treatment efficacy appear to be robust and generally applicable across the demographic groups tested.

### 3.4. Consumer Preference Segmentation

To explore heterogeneity in consumer preferences, a hierarchical cluster analysis was performed on the Overall Liking scores. This method groups consumers into distinct segments based on their preference patterns across the three apple treatments. The optimal number of clusters was determined by quantitatively assessing the results from the silhouette and “elbow” (within-cluster sum of squares) methods (d).

**Figure 5:**
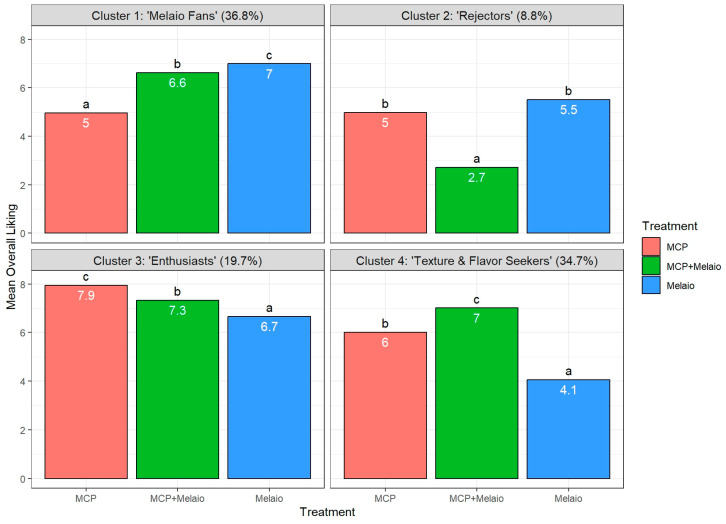
**Consumer preference segments for ‘Annurca’ apples.** The four panels show the mean Overall Liking Scores for distinct consumer segments identified through hierarchical cluster analysis. Each panel is labeled with its cluster number, a descriptive name, and its size as a percentage of the total consumer panel. Within each panel, the bars represent the mean liking for the three postharvest treatments: the ‘MCP’ treatment, the integrated ‘MCP+Melaio’ treatment, and the traditional ‘Melaio’ processing. Liking was rated on a 9-point hedonic scale (1 = extremely dislike, 9 = extremely like), and the specific mean scores are displayed within each bar. Different lowercase letters above the bars indicate statistically significant differences (*p* < 0.05) among the treatments within that consumer segment.

Based on these methods four distinct consumer segments were identified (Figure 5).

A Chi-squared analysis was performed to determine if demographic factors were related to cluster membership. The results indicated that a consumer’s age group did not significantly predict which preference cluster they belonged to (χ^2^(3) = 7.07, *p* = 0.07). Similarly, consumer gender showed no significant relationship with cluster membership (χ^2^(3) = 1.14, *p* = 0.77). This suggests the distribution of both age and gender was consistent across all segments (Supplementary Table S2).

The identified consumer segments are Cluster 1: “Melaio Fans” (37% of consumers). The largest segment (*n* = 179) demonstrated a clear preference hierarchy, rating all three treatments significantly different from one another. Their highest preference was for the traditional ‘Melaio’ treatment (mean liking = 7.0), followed by the integrated ‘MCP+Melaio’ treatment (6.6). They significantly disliked the ‘MCP’ treatment (5.0). The sensory data reveal that this preference is flavor-driven; this group penalized the ‘MCP’ apple for its low Sweet Taste (4.12) and high Acidity (5.15), while being the only group not to perceive the traditional ‘Melaio’ apple as overly mealy (4.67). This cluster had the highest percentage of younger consumers (89%). However, while groups showed some differences in age distribution, a Chi-squared analysis revealed no statistically significant association between cluster membership and age group (χ^2^(3) = 7.07, *p* = 0.07). Cluster 2: “MCP+Melaio Rejectors” (9% of consumers). This was the smallest (*n* = 43) but most polarized segment. These consumers showed no statistical difference in their liking for the ‘Melaio’ (5.8) and ‘MCP’ (5.3) treatments. However, they strongly and significantly rejected the integrated ‘MCP+Melaio’ treatment, giving it an extremely low score (3.0). The sensory analysis explains this unique aversion: this was the only group to perceive the ‘MCP+Melaio’ apple as having exceptionally low Juiciness (4.44) and Sweet Taste (3.60), resulting in a profile they found uniquely undesirable. Cluster 3: “Enthusiasts” (20% of consumers). This segment (*n* = 96) can be characterized as apple enthusiasts, giving high liking scores to all three treatments, which were all statistically different from each other (‘MCP’ > ‘MCP+Melaio’ > ‘Melaio’). Their top preference for the ‘MCP’ apples (7.9) is directly explained by their perception of their superior texture; they gave it the highest Crunchiness (7.14) and lowest Mealiness (3.16) scores of any group for any apple. This group also had the highest proportion of female participants (41%). Cluster 4: “Texture & Flavor Seekers” (35% of consumers). The large segment (*n* = 169) demonstrated a highly significant preference hierarchy (‘MCP+Melaio’ > ‘MCP’ > ‘Melaio’). Their strong dislike for the traditional ‘Melaio’ apple (4.1) is directly attributable to its perceived poor texture; they rated it as the most mealy (5.72) and least crunchy (3.56) of all samples. The ‘MCP+Melaio’ treatment produced their ideal apple because it combined the best of both worlds: it maintained the excellent texture of the ‘MCP’ treatment (low Mealiness of 2.98) while also developing a significantly higher Sweet Taste (5.70) than the ‘MCP’ apple alone (4.52). This cluster contained the highest proportion of older consumers (15%).

### 3.5. Sensory Drivers of Cluster Preferences

**Figure 6:**
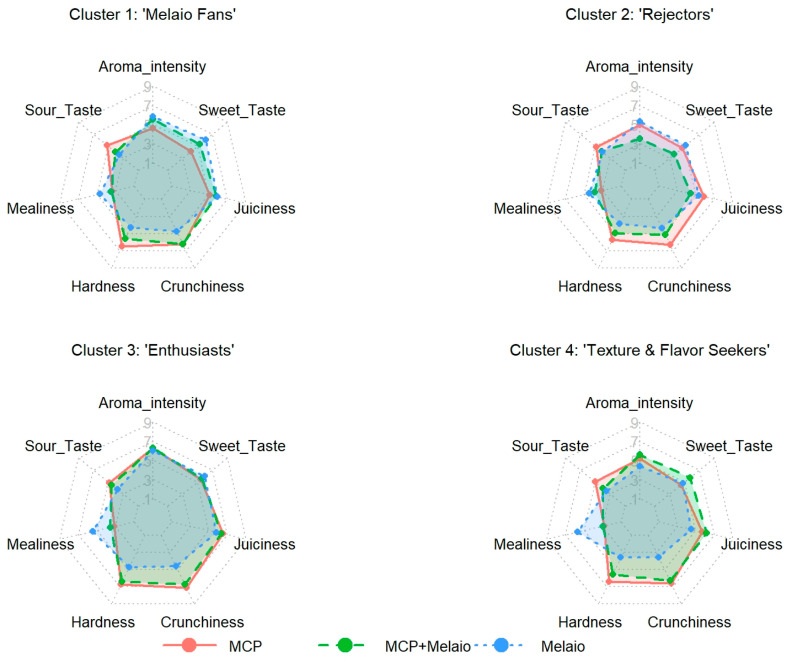
**Mean sensory profiles of the three apple treatments as perceived by each consumer segment.** Radar charts show mean intensity scores (1–9) for six attributes: Hardness, Crunchiness, Juiciness, Sweetness, Sourness, and Mealiness. Colored lines represent treatments: ‘MCP’ (salmon), ‘MCP+Melaio’ (green), and ‘Melaio’ (blue).

To understand the underlying reasons for the different preference patterns, the sensory attribute ratings were analyzed for each consumer segment (Figure 6 and Supplementary Table S3).

This revealed that the clusters had distinctly different sensory perceptions of the apples, which directly explains their liking scores. The “Texture & Flavor Seekers” (Cluster 4), who strongly preferred the ‘MCP+Melaio’ apples, clearly prioritized texture. They perceived the traditional ‘Melaio’ apple as significantly more mealy (5.72) and less crunchy (3.56) than any other group. The integrated ‘MCP+Melaio’ treatment produced their most-liked apple because it solved this textural issue (Mealiness = 2.98) while also developing a much higher Sweet Taste (5.70) compared to the ‘MCP’ treatment (4.52). Conversely, the “Melaio Fans” (Cluster 1) were primarily driven by flavor. They were the only group that did not perceive the ‘Melaio’ apple as particularly mealy (4.67) and instead penalized the ‘MCP’ apple for its low Sweet Taste (4.12) and high acidity (5.15). Their preference for the two treatments involving the *melaio* process suggests an appreciation for the characteristic flavor that develops during traditional reddening. The preferences of the “Enthusiasts” (Cluster 3) were driven by their perception of superior quality across all treatments. They gave the highest scores for key positive attributes to their preferred samples, rating the ‘MCP’ apple as the crunchiest (7.14) and the ‘Melaio’ apple as the sourest (6.01) and sweetest (5.95). Finally, the unique aversion of the “Rejectors” (Cluster 2) to the ‘MCP+Melaio’ treatment is explained by their sensory data. They were the only group to perceive this apple as having exceptionally low juiciness (4.44) and a very low Sweet Taste (3.60), resulting in a profile they found uniquely undesirable. These results demonstrate that consumer heterogeneity in liking is directly linked to differences in how key sensory attributes are perceived and weighted by different segments of the population.

### 3.6. Drivers of Liking for Each Consumer Segment

**Figure 7:**
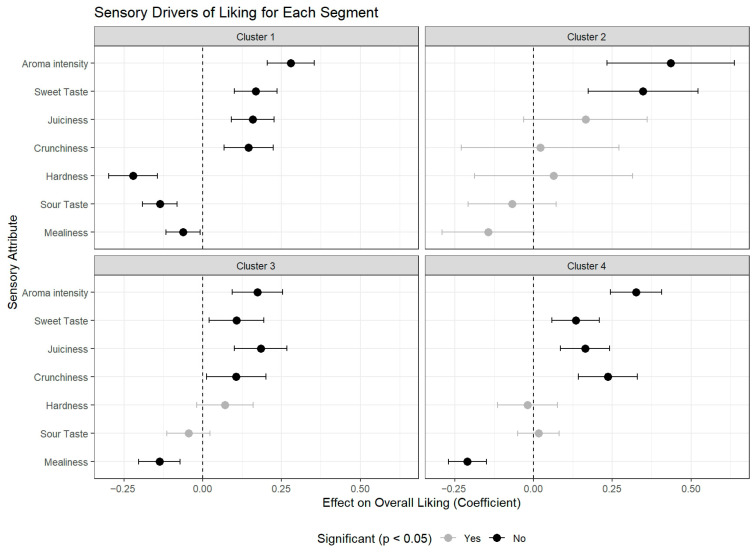
**Sensory drivers of liking for each consumer segment.** The plot shows coefficient estimates from four linear mixed-effects models (one per cluster) modeling Overall Liking as a function of sensory attributes. Points indicate the estimated effect of a one-unit increase in each attribute on the liking score. Positive values denote positive drivers of liking; negative values denote negative ones. Black dots indicate significant effects (*p* < 0.05), grey dots are non-significant.

To provide a statistical confirmation of the sensory factors motivating each consumer segment, a separate linear mixed-effects model was built for each cluster to identify its specific drivers of liking (Figure 7 and Supplementary Table S4). The models confirmed that the segments prioritize sensory attributes differently, providing a quantitative explanation for their distinct preference patterns.

For the ‘Melaio Fans’ (Cluster 1), the model revealed a highly defined profile where nearly all attributes were significant. The strongest positive driver was Aroma Intensity (β = +0.28, *p* < 0.001), but Sweet Taste (β = +0.17, *p* < 0.001), Juiciness (β = +0.16, *p* < 0.001), and Crunchiness (β = +0.15, *p* < 0.001) also significantly increased liking. Conversely, this group was penalized by negative attributes, with Hardness (β = −0.22, *p* < 0.001) and Sour Taste (β = −0.14, *p* < 0.001) being highly significant negative drivers, alongside a smaller but significant dislike for Mealiness (β = −0.06, *p* < 0.05). This provides strong statistical evidence that this group’s preference is for a classic, high-quality apple that is sweet, aromatic, and texturally sound.

In contrast, the model for the ‘Enthusiasts’ (Cluster 3) showed an appreciation for flavor and texture, with Aroma Intensity (β = +0.17, *p* < 0.001) and Juiciness (β = +0.18, *p* < 0.001) as the primary positive drivers, followed by Sweet Taste (β = +0.11, *p* < 0.05) and Crunchiness (β = +0.11, *p* < 0.05). Their only significant dislike was for Mealiness (β = −0.14, *p* < 0.001). Unlike Cluster 1, Hardness and Sour Taste were not significant factors, reinforcing their profile as a group that appreciates high-quality apples but is more tolerant of certain textures and flavors.

The profile for ‘Texture & Flavor Seekers’ (Cluster 4) lived up to its name. Aroma Intensity (β = +0.33, *p* < 0.001) was the most powerful positive driver overall, followed by strong textural drivers like Crunchiness (β = +0.24, *p* < 0.001) and Juiciness (β = +0.16, *p* < 0.001), and Sweet Taste (β = +0.13, *p* < 0.001). The strongest negative predictor was Mealiness (β = −0.21, *p* < 0.001). This confirms their preference is governed by the joint pursuit of a crisp, non-mealy texture and a sweet, aromatic flavor profile.

Finally, the model for the small ‘Rejectors’ (Cluster 2) was the most distinct. The only significant drivers of liking were Aroma Intensity (β = +0.44, *p* < 0.001) and Sweet Taste (β = +0.35, *p* < 0.001). The lack of significance for any textural attributes (Hardness, Crunchiness, Juiciness, Mealiness) suggests this group’s preferences are less influenced by texture and are instead driven primarily by specific flavor and aroma notes.

## 4. Discussion

This study demonstrated that the strategic integration of a modern postharvest technology with a traditional practice can significantly improve the consumer acceptability of ‘Melannurca Campana’ PGI apples. The primary finding was that the combined ‘MCP+Melaio’ treatment yielded apples that were significantly more liked by consumers than those from either the traditional ‘Melaio’ process or a 1-MCP treatment alone. This conclusion is based on the synergistic effect of a balanced approach that simultaneously optimizes the fruit’s texture, flavor, and, most critically, its aromatic profile. Moreover, our consumer segmentation analysis revealed that this overall preference is not monolithic. Instead, it is driven by the combined treatment’s unique ability to satisfy the distinct sensory priorities of multiple, large consumer segments, particularly the “Texture & Flavor Seekers” who represent over a third of the panel [19].

Our sensory results confirmed the fundamental challenges of the traditional Annurca production system, as outlined in the literature [20]. The ‘Melaio’ process, while essential for developing the characteristic skin color and contributing to flavor, proved to be detrimental to fruit texture. Apples from this control group scored highest in undesirable Mealiness and lowest in Crunchiness. Our findings indicate that this loss of texture is so pronounced that it offsets the advantages of the fruit’s otherwise well-developed sweetness and aroma, yielding an overall liking score comparable to that of the texturally superior but aromatically bland 1-MCP apples. For instance, this was particularly evident in the large ‘Texture & Flavor Seekers’ segment, who severely penalized the ‘Melaio’ apple for its perceived texture, rating it as the mealiest (5.72) and least crunchy (3.56) of all samples.

In contrast, the treatment involving only 1-MCP produced an apple with an excellent textural profile, characterized by the highest Crunchiness and lowest Mealiness scores. This preservation of texture is consistent with the known effects of 1-MCP in slowing senescence-related softening [21,22]. However, the 1-MCP ethylene inhibition, while beneficial for maintaining texture, was associated with a less favorable flavor and aroma profile. The ‘MCP’ apples were rated as having the lowest Sweet Taste and, critically, the lowest Aroma Intensity. This strongly suggests that by comprehensively arresting ethylene action, 1-MCP also halted the downstream metabolic pathways responsible for starch-to-sugar conversion [23] and the biosynthesis of volatile organic compounds that constitute fruit aroma [24]. The result was a texturally improved but organoleptically incomplete fruit. This flavor deficiency was most strongly rejected by the “Melaio Fans” segment (37% of consumers), who heavily penalized the ‘MCP’ apple for its low Sweet Taste (4.12) and high acidity.

The effectiveness of the integrated ‘MCP+Melaio’ treatment lies in its ability to precisely mitigate the weaknesses of the other two approaches. The initial 1-MCP application appears to have acted as a “textural protectant”, establishing a firm, non-mealy foundation by blocking the initial, most intense phase of ethylene-induced softening. This effect is consistent with long-term storage studies showing that 1-MCP significantly preserves tissue firmness [25]. Notably, this more compact structure was also associated with reduced emission of volatile organic compounds (VOCs), confirming that 1-MCP not only delays softening but also modulates aroma development by limiting the ethylene-dependent pathways involved in VOC biosynthesis and diffusion [25]. This intervention preserved the structural integrity of the fruit, upon which the subsequent traditional *melaio* reddening process could proceed. We hypothesize that this period allowed for a partial recovery of ethylene synthesis or receptor activity, sufficient to trigger the metabolic pathways for flavor and aroma development without causing runaway textural collapse [9,26]. This explanation is strongly supported by our data. The ‘MCP+Melaio’ apples achieved the highest Aroma Intensity score, an attribute our model identified as the single most important driver of consumer preference. The resulting fruit successfully combined this enhanced aromatic complexity with desirable sweetness and superior texture. This created a comprehensive and balanced sensory profile that directly explains its significantly higher overall acceptability. This balanced profile appealed to both the “Texture & Flavor Seekers”, who valued its excellent texture, and the “Melaio Fans”, who appreciated its recovered flavor profile, thus satisfying the two largest and otherwise conflicting consumer groups. Furthermore, the sensory profiles revealed an interesting feature of the consumer segmentation. The shape of the sensory profile for the traditional ‘Melaio’ apple was highly consistent across all segments. This suggests that the ‘Melaio’ apple acts as a stable sensory anchor; consumers agree on its core attributes even if their hedonic ratings differ. Conversely, the novel treatments, particularly the ‘MCP+Melaio’ apple, elicited highly heterogeneous sensory perceptions. This perceptual divergence highlights that the sensory identity of a novel product is not fixed but is interpreted differently by consumer segments based on their unique priorities. The success of the integrated treatment was therefore not just in creating a product with a preferred sensory profile, but in creating a profile that could be positively interpreted by multiple, distinct consumer groups.

Furthermore, our analysis of the storage time revealed a highly significant interaction with the postharvest treatments, providing critical insights into the sensory shelf-life of the apples [25,27]. The consumer’s liking for apples from the traditional ‘*melaio*’ process declined at a considerably faster rate over the storage period compared to the two treatments involving the application of 1-MCP. This rapid sensory decay represents a substantial economic risk for producers, limiting marketability and potentially increasing post-harvest losses. In contrast, the stable, high-quality sensory profile of the ‘MCP+Melaio’ apples should have a positive impact on the shelf-life. This finding is of considerable practical importance, as it not only addresses the initial textural deficit but also ensures a more consistent premium product over a longer commercial window, a key factor for market success. 

The traditional *melaio* system, while culturally significant, poses a considerable economic risk to growers due to quality loss and inconsistency. Our results propose a scientifically validated strategy that integrates a modern, commercially available tool (1-MCP) into the traditional production framework. This approach respects the PGI protocol’s requirement for open-air reddening while simultaneously addressing its main drawback, the rapid textural deterioration. By delaying ethylene-mediated softening, 1-MCP effectively acts as a “textural protectant”, supporting higher pack-out rates, extending market availability, and ensuring a more consistent, premium product with enhanced sensory quality that may foster consumer preference and loyalty. A similar outcome has been observed beyond our case study. Preharvest application of 1-MCP in the traditional Polish cultivar ‘Szampion’ allowed delayed harvesting without quality loss, and consistently maintained higher firmness and titratable acidity during storage [28]. These findings reinforce the versatility of 1-MCP as a tool for integrating modern postharvest science into traditional systems, ensuring quality preservation without compromising authenticity.

While this study provides valuable insights, it also opens avenues for future research. The consumer panel was deliberately recruited from a single region in Italy, where familiarity with the characteristic taste profile of ‘Annurca’ apples strengthens the relevance of the findings for the PGI consortium. This regional recruitment strategy aligns with previous evidence indicating that consumers tend to select apples based on prior experiences with sensory and internal quality attributes and localized preferences, which may differ from more generalized consumer trends [29]. Our demographic analysis revealed no statistically significant association between preference patterns and either age or gender. However, the trend approaching significance for age, combined with the study’s relatively coarse age dichotomy, suggests that a more extensive investigation with finer demographic stratification could yield deeper insights. This is supported by previous segmentation work using PCA, which showed that apple sensory preferences vary markedly by age group: younger consumers prioritize taste, size, and sweetness; middle-aged consumers focus more on firmness, juiciness, and flavor; and older consumers value color, firmness, and appearance [30]. These differentiated sensory priorities may underlie nuanced age-related preference patterns that our current study design could not fully resolve. On the other hand, our sample largely represents the preferences of the Millennial and Gen Z cohorts. While this provides valuable, forward-looking insights for the ‘Annurca’ PGI consortium regarding its future consumer base, it also limits the generalizability of our findings to older demographics. Furthermore, given that Aroma Intensity emerged as a key driver of liking, future research may be important to identify the specific volatile compounds that differentiate the treatments [31]. While our analysis identified the key drivers of liking, alternative approaches could employ Just-About-Right scales and penalty analysis to provide a more direct diagnosis of attribute optimality and guide further product optimization [32]. Finally, although the findings support the integration of 1-MCP into the ‘Annurca’ postharvest system, large-scale adoption may be constrained by the additional treatment costs and the requirement for approval from the ‘Melannurca Campana’ PGI consortium. Future research should include an economic assessment and collaborative efforts with the consortium to facilitate the implementation of this practice.

## 5. Conclusions

This work demonstrates that tradition and technology can be effective partners. By strategically applying 1-MCP prior to the traditional reddening process, it is possible to produce a ‘Melannurca Campana’ apple with high aroma and flavor intensity, alongside improved textural consistency. This integrated postharvest system offers a practical, effective, and scientifically grounded solution to a long-standing challenge, providing a pathway to enhance the quality, consistency, and economic value of this unique and culturally important fruit.

## Figures and Tables

**Table 1:** Mean sensory scores for apples subjected to different postharvest treatments. Different letters within a row indicate significant differences among treatments (*p* < 0.05) according to Tukey’s HSD test.

Sensory Attribute	MCP	MCP+Melaio	Melaio
***Flavor attributes***			
Aroma Intensity	5.15 a	5.54 b	5.35 ab
Sweet Taste	4.52 a	5.31 b	5.60 c
Sour Taste	5.14 c	4.23 b	3.64 a
***Texture attributes***			
Crunchiness	6.62 c	6.29 b	4.36 a
Juiciness	5.72 b	5.94 b	5.40 a
Mealiness	3.12 a	3.38 a	5.19 b
Hardness	6.44 c	5.68 b	4.16 a

## Data Availability

The original contributions presented in the study are included in the article/Supplementary material, further inquiries can be directed to the corresponding author.

## Supplementary Materials

The following supporting information can be downloaded at https://www.mdpi.com/article/10.3390/foods14203554/s1. Supplementary Table S1. Pairwise statistical comparisons for the three postharvest apple treatments. Supplementary Table S2. Demographic characterization of the four consumer segments identified through cluster analysis. Supplementary Table S3. Mean sensory attribute ratings for the three postharvest treatments as evaluated by consumers within each of the four identified clusters. Supplementary Table S4. Sensory drivers of liking for each consumer segment. Supplementary Figure S1: Determination of the optimal number of clusters (k) for k-means clustering using the Silhouette and Elbow (WSS) methods.
