# Supplementary material for: Resolving the Texture–Flavor Trade-Off in ‘Annurca’ Apples with an Integrated Postharvest System
Source: Foods. 2025 Oct 18;14(20):3554. doi: 10.3390/foods14203554 (PMC12564671; doi:10.3390/foods14203554)
Supplement: Supplementary file 1 [file foods-14-03554-s001.zip › Supplementary Material.pdf]

Supplementary Material

Supplementary Table S1. Pairwise statistical comparisons for the three postharvest apple treatments.

Treatment comparison	Estimate of difference	SE of difference	t-ratio	p-value
**MCP+Melaio vs. Melaio**	+0.699	0.105	6.64	< 0.001
**MCP+Melaio vs. MCP**	+0.702	0.106	6.63	< 0.001
**Melaio vs. MCP**	-0.003	0.103	-0.03	0.999

Supplementary Table S2. Demographic characterization of the four consumer segments identified through cluster analysis. The clusters were derived from a hierarchical cluster analysis based on individual consumer overall liking scores for the three apple treatments. Size refers to the total number of consumers assigned to each cluster. Percentages for gender (Female, Male) and age group (Younger ≤ 30 years, Older > 30 years) are calculated based on the total size of each cluster. The sum of percentages for a given demographic may be less than 100% due to missing data from participants who did not provide this information.

Cluster	Size	Gender		Age	
		Female (%)	Male (%)	Younger (%)	Older (%)
**1**	179	34.6	61.5	88.8	6.7
**2**	43	37.2	58.1	81.4	11.6
**3**	96	40.6	55.2	87.5	8.3
**4**	169	37.9	56.8	79.3	14.8

Supplementary Table S3. Mean sensory attribute ratings for the three postharvest treatments as evaluated by consumers within each of the four identified clusters.

Cluster	Treatment	Aroma Intensity	Sweet Taste	Sour Taste	Crunchiness	Juiciness	Mealiness	Hardness
**1**	MCP	4.61	4.12	5.15	6.26	5.13	3.37	6.47
**1**	MCP+Melaio	5.55	5.29	4.02	6.22	5.80	3.51	5.60
**1**	Melaio	5.81	6.06	3.53	4.79	5.93	4.67	4.33
**2**	MCP	4.98	4.67	4.86	6.33	5.88	3.17	5.70
**2**	MCP+Melaio	3.49	3.60	4.12	5.12	4.44	3.85	4.95
**2**	Melaio	5.30	5.12	4.02	4.37	5.36	4.47	3.88
**3**	MCP	6.31	5.45	4.83	7.14	6.53	3.16	6.73
**3**	MCP+Melaio	6.28	5.60	4.54	6.71	6.37	3.59	6.40
**3**	Melaio	6.01	5.95	3.72	4.62	5.83	5.46	4.73
**4**	MCP	5.21	4.52	5.01	6.60	5.70	2.86	6.40
**4**	MCP+Melaio	5.60	5.70	3.97	6.30	6.15	2.98	5.56
**4**	Melaio	4.40	4.75	3.49	3.56	4.49	5.72	3.60

Supplementary Table S4. Sensory drivers of liking for each consumer segment. The values in the table are the standardized regression coefficients (β) from separate linear mixed-effects models built for each cluster, with Overall Liking as the dependent variable. A positive coefficient indicates that an increase in the intensity of the attribute leads to a higher liking score, while a negative coefficient indicates the opposite. The statistical significance of each coefficient is indicated by asterisks (* $p < 0.05$, ** $p < 0.01$, *** $p < 0.001$).

Attribute	Cluster 1	Cluster 2	Cluster 3	Cluster 4
Hardness	-0.22***	0.06	0.07	-0.02
Crunchiness	0.15***	0.02	0.11*	0.24***
Juiciness	0.16***	0.17	0.18***	0.16***
Mealiness	-0.06*	-0.14	-0.14***	-0.21***
Sweet Taste	0.17***	0.35***	0.11*	0.13***
Aroma Intensity	0.28***	0.44***	0.17***	0.33***
Sour Taste	-0.14***	-0.07	-0.05	0.02

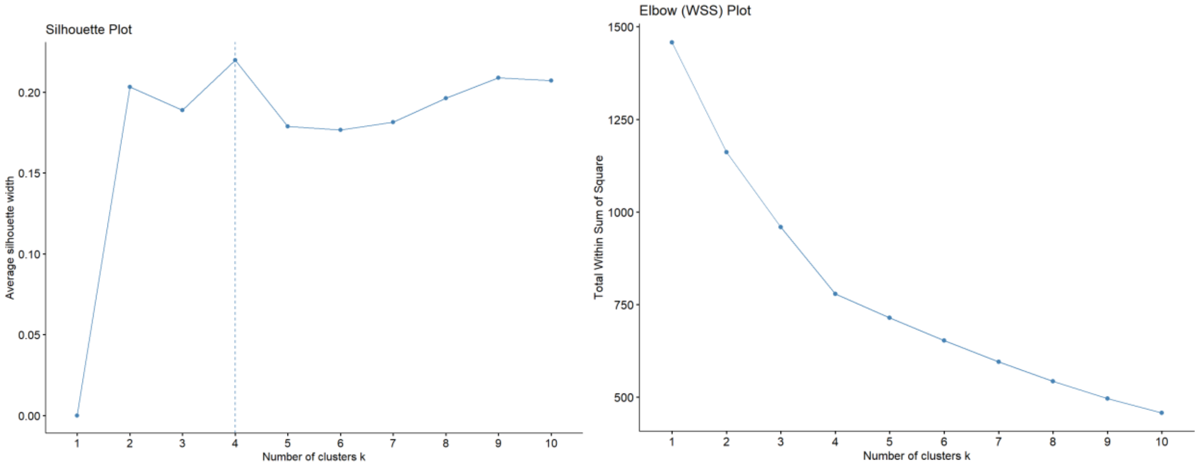

Supplementary Figure S1: Determination of the optimal number of clusters (k) for k-means clustering using the Silhouette and Elbow (WSS) methods. (Left Panel) The Average Silhouette method. The plot shows a clear peak at k=4 (indicated by the vertical dashed line), suggesting it as the optimal choice for cluster separation. (Right Panel) The Elbow method plots the total within-cluster sum of squares (WSS) as a function of the number of clusters (k). The optimal k is located at the "elbow" of the curve, representing the point where adding another cluster no longer provides a substantial reduction in WSS. In this plot, an elbow is more clearly observed around k=4.
